# Supplementary material for: Exploring context for implementation of inclusive education for children with developmental disabilities in mainstream primary schools in Ethiopia
Source: PLoS One. 2024 Aug 9;19(8):e0307576. doi: 10.1371/journal.pone.0307576 (PMC11315310; doi:10.1371/journal.pone.0307576)
Supplement: S1 File — Topic Guides Used For Interviews. (DOCX) [file pone.0307576.s001.docx]

**S1 File: Topic Guides**

Topic Guides Used For Interviews

**Interview Guide for General Informants**

- What does “inclusive education” mean to you?
- What are the difficulties presented by children with disabilities?
- What are your views on the policy about including children with disabilities in mainstream classrooms, alongside other children?
- What are your views on including children with developmental disorders in mainstream classrooms in the Ethiopian setting (for example children with social and communication impairments, or with emotional, behavioural or cognitive challenges)?
- Do you think there can be negative effects of including children with developmental disorders in mainstream classrooms, alongside other children?
- What are the positive aspects of including children with developmental disorders in mainstream classrooms, alongside other children?
- Can you think of any examples where children with developmental disorders receive appropriate education?
- And can you think of any examples where children with developmental disorders are successfully included in mainstream schools?
- Why are many children with developmental disorders enrolled in special schools?
- Why are many children with developmental disorders excluded from education?
- What would make it easier to enrol children with developmental disorders in mainstream schools?
- What, if anything, are you aware of that is being done in Addis Ababa to influence this situation?
- What would make it easier for children with developmental disorders to be included and accepted by their teachers and their peers in school?
- Is there anything else that you would like to share?

***Additional Questions for SEN Experts/Academics (Including NGO Representatives)***

- Could you describe how teachers in mainstream schools teach children with developmental disorders in the classroom, as far as you are aware?
- Could you describe how teachers keep the children with developmental disorders in the classroom when they are enrolled in mainstream schools?
  - What usually are teachers’ attitudes to including these children?
  - Is there anyone who disapproves?
- What do you think should be done when including children with developmental disorders in mainstream schools?
- Can you describe what education looks like for children with developmental disorders in Addis Ababa?

***Additional Questions for Clinicians***

- Do the children with developmental disorders that attend your clinic go to school? Where?
- As far as you are aware, for example from stories of children attending your clinic, how are children with developmental disorders taught and treated when they are enrolled in mainstream schools?
- What do you think should be done when including children with developmental disorders in mainstream schools?

***Additional Questions for Policy-makers***

- Could you describe how teachers keep the children with developmental disorders in the classroom when they are enrolled in mainstream schools?
  - What usually are teachers’ attitudes to including these children?
  - Is there anyone who disapproves?
- Can you describe what education looks like for children with developmental disorders in Addis Ababa?

**Interview Guide for Teachers and Principals**

- What does “inclusive education” mean to you?
- Are there any children with disabilities/difficulties enrolled in your school? And in your classroom?
  - If so, ask the following 2 questions:
    - How would you describe their difficulties?
    - Are there any children who have slow development, difficulties in learning or communicating, or behavioural and emotional challenges?
  - Yes -> ask questions 1-15
  - No -> ask questions a-j

***YES: for Schools Attended by Children With Developmental Delays***:

1. What are your views on including children with disabilities in mainstream classrooms, alongside other children?
2. What about including children who develop slowly or who have emotional and behavioural challenges?
3. Do you think there can be negative effects of including children with developmental disorders in mainstream classrooms, alongside other children?
4. What are the positive aspects of including children with developmental delays in mainstream classrooms, alongside other children?
5. What are the challenges in including children who develop slowly or have emotional and behavioural challenges in mainstream schools?
6. Could you explain what happens when a child with developmental delays enrols in this school?
7. What supports the enrolment of children with developmental delays and helps to overcome the challenges?
8. What else could make it easier to enrol children with developmental delays in mainstream schools?
9. How is it possible for you/teachers in this school to keep children with developmental disorders in the classroom?
   1. What are your/other teachers’ attitudes to including these children?
   2. Is there anyone who disapproves?
10. Could you describe how you/teachers in this school teach children with developmental delays in the classroom?
11. Do you/teachers in this school experience any challenges when there are children with developmental delays in the classroom?
12. Who do you think should support inclusive education?
13. What support is available from national and local governments?
14. Are there any additional unmet needs?
15. [ADDITIONAL QUESTION FOR PRINCIPALS]: What is your role, as a principal in an inclusive school?
16. Is there anything else that you would like to share?

***No: for Schools not Attended by Children With Developmental Delays:***

1. What are your views on including children with disabilities in mainstream classrooms, alongside other children?
2. What about including children who develop slowly or have emotional and behavioural challenges?
3. Do you think there can be negative effects of including children with developmental disorders in mainstream classrooms, alongside other children?
4. What are the positive aspects of including children with developmental delays in mainstream classrooms, alongside other children?
5. What are the challenges in including children who develop slowly or have emotional and behavioural challenges in mainstream schools?
6. Could you explain what would happen if a child with developmental delays enrolled in this school?
7. What would make it easier to enrol children with developmental delays in mainstream schools?
8. Who do you think should support inclusive education?
9. What support may be needed from national and local governments?
10. Is there anything else that you would like to share?

**Interview Guide for Caregivers**

- Can you tell me about your child?
- When and how did you know that he/she had a developmental delay?
- Is your child enrolled in a school?
  - Yes -> ask questions 1-20
  - No -> ask questions a-l

***YES: for Caregivers of Children Enrolled:***

1. Is he/she in a special school or mainstream school? Why?
2. [IF IN MAINSTREAM SCHOOL]: Is he/she in a special unit or a mainstream classroom?
3. When did he/she start going to school?
4. Did you face any challenges in enrolling your child in school? Can you explain the challenges?
5. What made it possible to enrol your child?
6. Are you happy about your child’s education in this school?
7. What does “inclusive education” mean to you?
8. What are your views on including children who develop slowly in mainstream schools?
9. Do you think there can be negative effects of including children with developmental disorders in mainstream classrooms, alongside other children?
10. What are the positive aspects of including children with developmental delays in mainstream classrooms, alongside other children?
11. Could you describe the way in which your child is treated in the classroom, as far as you are aware?
12. Did you have any experiences when you felt your child was not treated properly?
13. What is going well with your child’s education?
14. What is not going well with your child’s education?
15. What do you think is your role as a caregiver in your child’s education?
16. Do you face any challenges in supporting you child’s education?
17. What support would you need? What would help you?
18. Is there anyone else that you feel should be involved in inclusive education?
19. After we have gathered enough information, our aim is to develop a training for teachers to help include children who develop slowly in mainstream classrooms and meet their needs. What would your child need from a similar intervention?
20. Is there anything else that you would like to share?

***No: for Caregivers of Children not Enrolled:***

1. Have you ever tried to enrol your child in a school?
2. [IF THEY HAVE TRIED]: What challenges did you face in enrolling your child?
3. What could have helped overcome these challenges?
4. Was he/she ever in school?
5. [IF THE CHILD WAS PREVIOUSLY ENROLLED]: Was he/she enrolled in a special or mainstream school? What happened next?
6. Would you prefer your child to be enrolled in a special or mainstream school? Why?
7. What does “inclusive education” mean to you?
8. What are your views on including children who develop slowly in mainstream schools?
9. Do you think there can be negative effects of including children with developmental disorders in mainstream classrooms, alongside other children?
10. What are the positive aspects of including children with developmental delays in mainstream classrooms, alongside other children?
11. After we have gathered enough information, our aim is to develop a training for teachers to help include children who develop slowly in mainstream classrooms and meet their needs. What would your child need from a similar intervention?
12. Is there anything else that you would like to share?
