# Supplementary material for: Exploring context for implementation of inclusive education for children with developmental disabilities in mainstream primary schools in Ethiopia
Source: PLoS One. 2024 Aug 9;19(8):e0307576. doi: 10.1371/journal.pone.0307576 (PMC11315310; doi:10.1371/journal.pone.0307576)
Supplement: S2 File — Summary of codes developed and applied in the analysis. (DOCX) [file pone.0307576.s002.docx]

**S2 File: Codebook**

Summary of codes developed and applied in the analysis.

| CONTEXT |
| --- |
| 1. GEOGRAPHICAL |
| Lack of accessibility of schools due to distance |
| Difficulty accessing transport to take child to school |
| Urban vs rural divide in community attitudes to DD |
| 2. EPIDEMIOLOGICAL |
| Acknowledging diversity of children with DD |
| Differences in support for different DDs |
| Behavioural disruptions of children with DD |
| Characteristics of children with DD |
| Children's medical comorbidities |
| Gender gaps |
| Education gap between TD girls and boys |
| Gender gap in diagnosis of autism |
| Gender inequalities in the community |
| Girls with DD additionally disadvantaged |
| Gender not a factor affecting inclusion of children with DD |
| Greater risk of sexual assault for girls |
| Impaired communication of children with DD |
| Increasing prevalence due to identification |
| Lack of information about prevalence of DD |
| Learning difficulties |
| Specific learning disability prevalence |
| Need for early intervention and inclusion |
| Need to address sensory sensitivity of children with DD |
| Recognition of potential of children with DD |
| Vulnerability to sexual assault |
| 3. SOCIO-ECONOMIC |
| MACRO |
| Context of low income |
| Lack of resources for education of TD children |
| Lack of and need for research on special education in Ethiopia |
| Current research on education of children with DD |
| Lack of overall services for children with DD |
| Lack of and need for support for employment of children with DD |
| Limited healthcare services |
| Lack of and need for accurate identification and assessment of DD |
| Experiences with DD diagnosis process |
| Lack of and need for assessment of DD severity |
| Lack of and need for early identification of DD |
| Screening tools not adapted to Ethiopian context |
| Lack of and need for healthcare accommodating DD |
| Lack of and need for specialists in DD |
| Perception of education in Ethiopia as exclusive |
| Lack of access to any school |
| Lack of access to education outside Addis Ababa |
| Rejection of children with DD from schools |
| Perception of good progress towards inclusive education currently |
| Actions encouraging and supporting schools to accept DD children |
| Potential for collaboration between government and NGOs |
| Reliance on international donors to fund inclusive education |
| MESO |
| Lack of and need for resources for inclusive education |
| Need for assistive devices |
| Need for teaching and learning materials |
| Need to build capacity of schools to support children with DD |
| Lack of and need for suitably trained teachers |
| Lack of capacity of special schools |
| MICRO |
| Caregiver strain for having DD child |
| Caregiver strain reduced by child's schooling |
| Caregiver strain worsened by child's schooling |
| Child is safer at school |
| Impact of parental SES on child treatment and schooling |
| Lack of support for children with DD in school |
| Need to support caregivers of children with DD |
| Caregivers supporting each other |
| Supporting caregivers financially where required |
| Supporting caregivers in registering child for school |
| Supporting caregivers to care for child |
| 4. SOCIO-CULTURAL |
| MACRO |
| Improved awareness recently |
| Lack of and need for awareness of DD in community |
| Lack of and need for awareness about potential of children with DD to learn |
| Negative attitude of community towards children with DD |
| Spiritual explanations for DD in community |
| Understanding of disability and impairment |
| Understanding of diversity among people in general |
| Zemi Yenus' legacy |
| MESO |
| Beliefs and attitudes within schools |
| Gender of staff may affect inclusion |
| Lack of and need for awareness of teachers about DD |
| Lack of understanding about multiple disabilities |
| Lower awareness of DD than sensory and physical disabilities |
| Negative attitude of teachers to children with DD |
| Lack of schools accepting children with DD |
| More inclusion of children with physical and sensory disabilities than DD |
| Sensory disabilities have less effect on cognitive ability |
| Negative attitudes of caregivers of TD children |
| Negative perception of children with DD as behaviourally challenging |
| Lack of and need for awareness of DD in healthcare |
| Need for clinicians to communicate diagnosis sensitively to caregivers |
| Stakeholder understanding of types of DD |
| MICRO |
| Children with DD not engaged in inclusive schools |
| Family beliefs and attitudes |
| Caregiver attitude and response to DD child |
| Caregiver response to DD diagnosis |
| Caregiver understanding of DD |
| Shame and internalised stigma |
| Children with DD kept at home |
| Family environment affecting access to DD services |
| Lack of and need for awareness of caregivers about DD |
| Lack of caregiver knowledge over where to take child for school |
| Siblings support siblings with DD |
| 5. POLITICAL |
| MACRO |
| Inclusion as a historical process |
| Lack of and need for awareness of government about DD |
| Lack of understanding about practical context |
| Limited number of SEN experts in institutions |
| Lack of and need for government attention to DD |
| Lack of and need for government attention to inclusive education |
| Lack of and need for government funding for inclusive education |
| Need for dedicated budget for special education |
| Need for budget for SEN teacher training |
| Lack of and need for government leadership and accountability |
| Need for accountability of government |
| Lack of funding due to lack of accountability |
| Limited extent of action for inclusive education |
| School mental health initiatives |
| Need for government attention to education in general |
| Need for separation between education and politics |
| Political situation in Ethiopia |
| Prioritising education for TD children over education for children with DD |
| MESO |
| Lack of and need for school leadership of inclusive education |
| Need for career structure for special needs teachers |
| Negative attitude of principals to children with DD |
| Need of principals positive attitude for growth of IE |
| Negative attitudes of regular teachers towards SEN teachers |
| 6. LEGAL |
| MACRO |
| Disability policy in Ethiopia |
| Lack of accountability for upholding rights of people with disabilities |
| No cohesive disability policy |
| Fragmented and inconsistent policies |
| Government not accountable for supporting people with disabilities |
| Schools not accountable for supporting children with DD |
| Some outdated policies use derogatory language |
| Right of children with DD to education not specified |
| Education policy in Ethiopia |
| Children must study grade levels at specific ages |
| Education and Training Policy |
| No focus on special needs education in policy |
| Signage of international conventions relating to education |
| Education Sector Development Program |
| Special education recognised in ESDP |
| Government policy for inclusive education |
| Difficult for schools to fulfill policy requirements |
| Difficulty with practical implementation of policy |
| Ineffectiveness of government policy for inclusive education |
| Need for clear policy for education of children with DD |
| Need for country wide policy implementation |
| Need for inclusion across society |
| Strategic plan for inclusive education |
| Focus more on integration than inclusion |
| Strengths of strategy |
| Ministry of Education directorate for special support |
| Developing strategy for inclusive education |
| Quality Improvement Program funding |
| Recent improvements in policy for education of people with disabilities |
| Change in law concerning students with disabilities in general education |
| Higher education provisions for students with disabilities |
| Recognition of sign language as a medium of instruction |
| Regional government determines extent of inclusive education |
| Schools cannot discriminate against children with DD |
| Special education unit within regional bureau |
| Subject-based teaching from grade 5 |
| Lack of and need for curriculum catering to children with DD |
| Resource Centers for special education |
| MESO |
| Medical reports needed for acceptance to special schools |
| 7. ETHICAL |
| Increasing recognition of rights of people with disabilities over time |
| Perception of right to education in general |
| Perception of inclusive education as a right |
| Need to recognise children with DD as people |
| Perception of need for inclusion in all aspects of schooling |
| Value of education |
| Education makes children learn and makes them active and happy |
| Potential of children with DD to contribute to society if supported |
| Schooling develops children's independence |
| SETTING |
| Lack of and need for accessible school environment |
| Safety concerns |
| Higher perceived risk of educating children with DD |
