# Supplementary material for: Exploring context for implementation of inclusive education for children with developmental disabilities in mainstream primary schools in Ethiopia
Source: PLoS One. 2024 Aug 9;19(8):e0307576. doi: 10.1371/journal.pone.0307576 (PMC11315310; doi:10.1371/journal.pone.0307576)
Supplement: Attachment — Submitted filename: Plos reviewed manuscript.pdf [file pone.0307576.s003.pdf]

PLOS ONE

Exploring context for implementation of inclusive education for children with developmental disabilities in mainstream primary schools in Ethiopia

--Manuscript Draft--

**Manuscript Number:**	PONE-D-23-36308
**Article Type:**	Research Article
**Full Title:**	Exploring context for implementation of inclusive education for children with developmental disabilities in mainstream primary schools in Ethiopia
**Short Title:**	Context for including children with developmental disabilities in mainstream schools in Ethiopia
**Corresponding Author:**	Elisa Genovesi<br>King's IoPPN: King's College London Institute of Psychiatry Psychology & Neuroscience<br>London, Greater London UNITED KINGDOM
**Keywords:**	autism; intellectual disabilities; Developmental disabilities; inclusive education; INCLUSION; Global Mental Health; Context; Africa; Ethiopia
**Abstract:**	A large gap in provision of services for children with developmental disabilities has been identified in Ethiopia, especially in the education system. Including children with disabilities in mainstream schools is encouraged by policies, but progress in this direction has been limited. This study aimed to explore stakeholders' perspectives on contextual factors relevant for inclusive education for children with developmental disabilities in mainstream schools in Addis Ababa, Ethiopia. Data were collected through semi-structured interviews with 39 local stakeholders, comprising caregivers of children with developmental disabilities, school teachers and principals/managers, non-governmental organisation representatives, government officials, clinicians and academics/consultants. We used template analysis to code the data and map them onto domains of the Context and Setting dimensions of the Context and Implementation of Complex Interventions framework (Pfadenhauer et al., 2017). Stakeholders discussed frameworks in the Legal and Ethical context endorsing the right of all children to education. However, they reported multiple reasons why children with developmental disabilities in Addis Ababa have limited access to education, either in special or mainstream schools. First, individual features, such as gender and support needs, discussed in the Epidemiological context, may affect the likelihood of a child with developmental disabilities to be accepted in school. Transportation challenges are a key barrier in the Geographical context. Socio-economic and Socio-cultural contexts present barriers at the levels of the nation, school and family, mostly related to limited services and material and financial resources and limited awareness of developmental disabilities. Stakeholders believe the currently limited but growing commitment in the Political context can support progress towards the removal of these barriers. Our findings can form the basis for development of an implementation plan that addresses such barriers and capitalises on existing facilitators.
**Order of Authors:**	Elisa Genovesi
	Ikram Ahmed
	Moges Ayele
	Winini Belay
	Olivia Burningham
	Amanda Chen
	Fikirte Girma
	Liya Tesfaye Lakew
	Charlotte Hanlon
	Rosa Anna Hoekstra

Powered by Editorial Manager® and ProduXion Manager® from Aries Systems Corporation

Additional Information:	
**Question**	**Response**
**Financial Disclosure**<br><br>Enter a financial disclosure statement that describes the sources of funding for the work included in this submission. Review the submission guidelines for detailed requirements. View published research articles from *PLOS ONE* for specific examples.<br><br>This statement is required for submission and **will appear in the published article** if the submission is accepted. Please make sure it is accurate.<br><br>**Funded studies**<br>Enter a statement with the following details:<br>• Initials of the authors who received each award<br>• Grant numbers awarded to each author<br>• The full name of each funder<br>• URL of each funder website<br>• Did the sponsors or funders play any role in the study design, data collection and analysis, decision to publish, or preparation of the manuscript?<br><br>Did you receive funding for this work?	Yes
Please add funding details.<br>as follow-up to "**Financial Disclosure**<br><br>Enter a financial disclosure statement that describes the sources of funding for the work included in this submission. Review the submission guidelines for detailed requirements. View published research articles from *PLOS ONE* for specific examples.<br><br>This statement is required for submission and **will appear in the published article** if the submission is accepted. Please make sure it is accurate.	The project has been funded by the UKRI Economic and Social Research Council through a London Interdisciplinary Social Science Doctoral Training Partnership (LISS DTP) studentship (ES/P000703/1), by the British Institute for Eastern Africa through a Thematic Grant and by undergraduate research project funding from King's College London. RAH, CH, FG and WB receive support from the National Institute for Health and care Research (NIHR200842) using UK aid from the UK Government and CH through the NIHR Global Health Research Group on Homelessness and Mental Health in Africa (NIHR134325) using UK aid from the UK Government. The views expressed in this publication are those of the authors and not necessarily those of the NIHR or the Department of Health and Social Care or other funders. CH receives support from the Wellcome Trust through grants 222154/Z20/Z and 223615/Z/21/Z.

**Funded studies**<br>Enter a statement with the following details:<br>• Initials of the authors who received each award<br>• Grant numbers awarded to each author<br>• The full name of each funder<br>• URL of each funder website<br>• Did the sponsors or funders play any role in the study design, data collection and analysis, decision to publish, or preparation of the manuscript?<br><br>Did you receive funding for this work?"	
Please select the country of your main research funder (please select carefully as in some cases this is used in fee calculation).<br>as follow-up to "**Financial Disclosure**<br><br>Enter a financial disclosure statement that describes the sources of funding for the work included in this submission. Review the submission guidelines for detailed requirements. View published research articles from *PLOS ONE* for specific examples.<br><br>This statement is required for submission and **will appear in the published article** if the submission is accepted. Please make sure it is accurate.<br><br>**Funded studies**<br>Enter a statement with the following details:<br>• Initials of the authors who received each award<br>• Grant numbers awarded to each author<br>• The full name of each funder<br>• URL of each funder website<br>• Did the sponsors or funders play any role in the study design, data collection and analysis, decision to publish, or preparation of the manuscript?<br><br>Did you receive funding for this work?"	UNITED KINGDOM - GB
**Competing Interests**	The authors have declared that no competing interests exist.

Powered by Editorial Manager® and ProduXion Manager® from Aries Systems Corporation

Use the instructions below to enter a competing interest statement for this submission. On behalf of all authors, disclose any competing interests that could be perceived to bias this work—acknowledging all financial support and any other relevant financial or non-financial competing interests.<br><br>This statement is required for submission and **will appear in the published article** if the submission is accepted. Please make sure it is accurate and that any funding sources listed in your Funding Information later in the submission form are also declared in your Financial Disclosure statement.<br><br>View published research articles from *PLOS ONE* for specific examples.<br><br>**NO authors have competing interests**<br><br>Enter: *The authors have declared that no competing interests exist.*<br><br>**Authors with competing interests**<br><br>Enter competing interest details beginning with this statement:<br><br>*I have read the journal's policy and the authors of this manuscript have the following competing interests: [insert competing interests here]*<br><br>* typeset	
**Ethics Statement**<br><br>Enter an ethics statement for this submission. This statement is required if the study involved:<br><br>• Human participants<br>• Human specimens or tissue<br>• Vertebrate animals or cephalopods<br>• Vertebrate embryos or tissues	Ethical approval was obtained from King's College London (RESCM-22/23-21930), the Scientific and Ethical Review Committee at CDT-Africa of Addis Ababa University and the Addis Ababa University College of Health Science Institutional Review Board (061/21/CDT). All participants provided written informed consent to participate.

Powered by Editorial Manager® and ProduXion Manager® from Aries Systems Corporation

• Field research<br><br>Write "N/A" if the submission does not require an ethics statement.<br><br>General guidance is provided below. Consult the submission guidelines for detailed instructions. **Make sure that all information entered here is included in the Methods section of the manuscript.**	

Powered by Editorial Manager® and ProduXion Manager® from Aries Systems Corporation

**Format for specific study types**<br><br>**Human Subject Research (involving human participants and/or tissue)**<br>• Give the name of the institutional review board or ethics committee that approved the study<br>• Include the approval number and/or a statement indicating approval of this research<br>• Indicate the form of consent obtained (written/oral) or the reason that consent was not obtained (e.g. the data were analyzed anonymously)<br><br>**Animal Research (involving vertebrate animals, embryos or tissues)**<br>• Provide the name of the Institutional Animal Care and Use Committee (IACUC) or other relevant ethics board that reviewed the study protocol, and indicate whether they approved this research or granted a formal waiver of ethical approval<br>• Include an approval number if one was obtained<br>• If the study involved *non-human primates*, add *additional details* about animal welfare and steps taken to ameliorate suffering<br>• If anesthesia, euthanasia, or any kind of animal sacrifice is part of the study, include briefly which substances and/or methods were applied<br><br>**Field Research**<br><br>Include the following details if this study involves the collection of plant, animal, or other materials from a natural setting:<br>• Field permit number<br>• Name of the institution or relevant body that granted permission	
**Data Availability**<br><br>Authors are required to make all data underlying the findings described fully available, without restriction, and from the time of publication. PLOS allows rare exceptions to address legal and ethical concerns. See the PLOS Data Policy and FAQ for detailed information.	No - some restrictions will apply

Powered by Editorial Manager® and ProduXion Manager® from Aries Systems Corporation

A Data Availability Statement describing where the data can be found is required at submission. Your answers to this question constitute the Data Availability Statement and **will be published in the article**, if accepted.<br><br>**Important:** Stating 'data available on request from the author' is not sufficient. If your data are only available upon request, select 'No' for the first question and explain your exceptional situation in the text box.<br><br>Do the authors confirm that all data underlying the findings described in their manuscript are fully available without restriction?	
**Describe where the data may be found in full sentences. If you are copying our sample text, replace any instances of XXX with the appropriate details.**<br><br>• If the data are **held or will be held in a public repository**, include URLs, accession numbers or DOIs. If this information will only be available after acceptance, indicate this by ticking the box below. For example: *All XXX files are available from the XXX database (accession number(s) XXX, XXX.).*<br>• If the data are all contained **within the manuscript and/or Supporting Information files**, enter the following: *All relevant data are within the manuscript and its Supporting Information files.*<br>• If neither of these applies but you are able to provide **details of access elsewhere**, with or without limitations, please do so. For example:<br><br>*Data cannot be shared publicly because of [XXX]. Data are available from the XXX Institutional Data Access / Ethics Committee (contact via XXX) for researchers who meet the criteria for access to confidential data.*<br><br>*The data underlying the results presented in the study are available from (include the name of the third party*	Data will be available upon request. The authors have carefully considered making the data available through a repository. However, in qualitative research and in global health this poses a series of issues, also recognised by evidence (e.g. Tsai et al., 2016). As such, the authors have agreed that it would be unethical to make data publicly available. First, quotes is the manuscript are carefully selected so as to not identify participants or link them to their personal data, but full transcripts may identify them, as professionals working on developmental disabilities in Addis Ababa are few and known to the population. The trascripts may also include personal data beyond participants' perspectives, such as their diagnosis or that of their children. Secondly, the data would largely not be available to researchers in Africa, who are disadvantaged when it comes to accessing publicly available data. Instead, it may be accessible to researchers in high-income countries who may be unfamiliar with the context, leading to potentially harmful interpretations of the data.

and contact information or URL).<br>• This text is appropriate if the data are owned by a third party and authors do not have permission to share the data.<br><br>* typeset	
Additional data availability information:	

Powered by Editorial Manager® and ProduXion Manager® from Aries Systems Corporation

Manuscript Click here to access/download;Manuscript;Exploratory study context manuscript.docx

Exploring context for implementation of inclusive education for children with developmental disabilities in mainstream primary schools in Ethiopia

Elisa Genovesi[1], Ikram Ahmed[2], Dr Moges Ayele[3], Winini Belay[4], Olivia Burningham[5], Amanda Chen[6], Dr Fikirte Girma[2], Liya Tesfaye Lakew[7], Prof Charlotte Hanlon[2,4,8], Dr Rosa A. Hoekstra[1]

1 Department of Psychology, Institute of Psychiatry, Psychology & Neuroscience, King's College London, London, UK

2 Department of Psychiatry, WHO Collaborating Centre for Mental Health Research and Capacity-Building, School of Medicine, College of Health Sciences, Addis Ababa University, Addis Ababa, Ethiopia

3 School of Psychology, College of Education and Behavioral Studies, Addis Ababa University, Addis Ababa, Ethiopia

4 Centre for Innovative Drug Development and Therapeutic Trials for Africa, Addis Ababa University, Addis Ababa, Ethiopia

5 Department of International Development, London School of Economics and Political Science, London UK

6 St Andrew's Mission School, Singapore

7 Nia Foundation Joy Center for Autism, Addis Ababa, Ethiopia

8 Centre for Global Mental Health, Department of Health Services and Population Research, Institute of Psychiatry, Psychology & Neuroscience, King's College London, London, UK

Corresponding Author: Elisa Genovesi; elisa.genovesi@kcl.ac.uk

Abstract

A large gap in provision of services for children with developmental disabilities has been identified in Ethiopia, especially in the education system. Including children with disabilities in mainstream schools is encouraged by policies, but progress in this direction has been limited. This study aimed to explore stakeholders' perspectives on contextual factors relevant for inclusive education for children with developmental disabilities in mainstream schools in Addis Ababa, Ethiopia. Data were collected through semi-structured interviews with 39 local stakeholders, comprising caregivers of children with developmental disabilities, school teachers and principals/managers, non-governmental organisation representatives, government officials, clinicians and academics/consultants. We used template analysis to code the data and map them onto domains of the Context and Setting dimensions of the *Context and Implementation of Complex Interventions* framework (Pfadenhauer et al., 2017). Stakeholders discussed frameworks in the Legal and Ethical context endorsing the right of all children to education. However, they reported multiple reasons why children with developmental disabilities in Addis Ababa have limited access to education, either in special or mainstream schools. First, individual features, such as gender and support needs, discussed in the Epidemiological context, may affect the likelihood of a child with developmental disabilities to be accepted in school. Transportation challenges are a key barrier in the Geographical context. Socio-economic and Socio-cultural contexts present barriers at the levels of the nation, school and family, mostly related to limited services and material and financial resources and limited awareness of developmental disabilities. Stakeholders believe the currently limited but growing commitment in the Political context can support progress towards the removal of these barriers. Our findings can form the basis for development of an implementation plan that addresses such barriers and capitalises on existing facilitators.

Introduction

Developmental disabilities (DD), including autism and intellectual disability, are characterised by difficulties or differences in cognitive ability, communication and social behaviour beginning in childhood (World Health Organization, 2016). They are a substantial cause of disability globally (Colombi & Ghaziuddin, 2017; Olusanya et al., 2018). However, services for children with DD are often limited, particularly in low- and middle-income countries, where most children with DD reside (Olusanya et al., 2018).

In Ethiopia, a very low-income country in Eastern Africa, services for children with DD are limited (Tekola et al., 2016) and often unknown to families who need them (Zeleke et al., 2018). The limited availability and access to services are aggravated by stigma towards children with DD (Side & Kumar, 2022; Tekola et al., 2020; Tilahun et al., 2016), which is largely unaddressed (Zeleke et al., 2018) and often leads to the social isolation of these children and their caregivers (Tekola et al., 2020). In a study surveying 102 caregivers of children with DD in the capital city, Addis Ababa, stigma was reported by the majority of respondents (Tilahun et al., 2016). Additionally, 47.1% of caregivers reported unmet needs in receiving professional support for their children with DD. The most prominent unmet needs of caregivers, reported by 74.5% of respondents, was access to appropriate education (Tilahun et al., 2016). In more recent qualitative studies, caregivers spoke of their wish for their children to be schooled (Szlamka et al., 2023; Zerihun et al., 2023). While promoting child development (Burningham, Chen, Genovesi et al., 2023), inclusion in education can also ensure that caring responsibilities are not placed exclusively on caregivers, as is often the case in Ethiopia (Side & Kumar, 2022). Based on a situational analysis of services for DD in the country (Tekola et al., 2016), a limited number of children with DD are enrolled in the few special schools located in Addis Ababa. A few inclusive education programmes are present in some mainstream schools (Tekola et al., 2016). However, these schools and programmes have long waiting lists and are not sufficient to accommodate all children with DD, who are often rejected from mainstream schools (Tekola et al., 2016).

The most recent Ethiopian Education Sector Development Programme (ESDP VI; Federal Democratic Republic of Ethiopia Ministry of Education, 2021) reported that education was provided to only 11% of all children with any special needs, although data specifically pertaining to children with DD were missing. The programme promotes inclusive education, also indicated by the governments endorsement of the United Nations (UN) Convention on the Rights of Persons with Disabilities (CRPD; UN General Assembly, 2007). However, progress towards inclusive education has proved difficult, mainly due to challenges in providing mainstream school teachers with appropriate resources and training (UN Committee on the Rights of Persons with Disabilities, 2016a; 2016b). Similar barriers were reported in a recent situational analysis of inclusive education in Ethiopia (Mergia, 2020), commissioned by UNESCO in preparation of the 2020 Global Education Monitoring Report, in qualitative and mixed-methods studies exploring teachers' perspectives in Addis Ababa (Schiemer, 2017), the northern region of Tigray (Franck & Joshi, 2017) and southern Ethiopia (Ginja & Chen, 2021).

These studies all considered children with any disabilities, rather than children with DD in particular. The complex needs of children with DD mean that they may experience higher exclusion rates than children with physical or sensory disabilities. For example, in Uganda, children with cerebral palsy and associated intellectual disability were four times less likely to attend school compared to those with cerebral palsy but no intellectual disability (Andrews et al., 2020). Further, a recent systematic review of 32 qualitative studies in

sub-Saharan Africa (Genovesi et al., 2022), highlighted that in multiple countries the few available special needs education training programmes for teachers gave them little or no information on how to address the needs of children with DD. In Ethiopia, the most recent “Master Plan” for the education of children with disabilities (Federal Democratic Republic of Ethiopia Ministry of Education, 2015b) focuses strongly on provisions and modifications for children with sensory and physical disabilities (e.g. teaching materials in Braille, sign language training for staff, ramps to access facilities, etc): relevant accommodations for children with DD are less prominent. Therefore, it is likely that the inclusion and quality education of children with DD in Ethiopia may be hindered by greater challenges than those reported in general disability studies (Franck & Joshi, 2017; Schiemer, 2017; Ginja & Chen, 2021).

A full picture of contextual factors and an understanding of local stakeholder perspectives on inclusive education is crucial so that strategies can be identified to improve educational access for children with DD in Ethiopia. In this study, we interviewed Ethiopian stakeholders to explore their perspectives on including children with DD in education in the capital city, Addis Ababa, with the goal to ultimately develop and implement a context-appropriate intervention. In a previous in-depth analysis of these data we focused on the potential benefits and drawbacks of inclusive education specifically for the development and inclusion iof children with DD (Burningham, Chen, Genovesi et al., 2023). We concluded that inclusive education can benefit children with DD, if potential drawbacks are managed, and as such is considered an acceptable goal to pursue. In the current report we present a comprehensive analysis of the same data, focusing not primarily on the children themselves, but on contextual factors relevant to the inclusion of children with DD in primary schools in Addis Ababa. This report and a companion paper reporting on implementation processes (Genovesi et al., 2023) together lead to a context-appropriate framework-based implementation model for educational inclusion of children with DD.

Stakeholders should be involved in all stages of intervention development and implementation (Pfadenhauer et al., 2017). One key contribution they can give is in identifying relevant contextual factors that can influence implementation: from macro-level factors—national regional; to meso-level factors—in communities and organisations, for example the school community; to micro-level factors—where interpersonal interactions occur, for example in families or between teacher and children (Pfadenhauer et al., 2017). As such, the views of stakeholders involved in the care of children with DD and in education provision in Ethiopia can shed light on contextual barriers and facilitators for promoting the education of children with DD in this context.

Specifically, in the analysis reported below, we investigated stakeholders’ perspectives on the existing education needs and challenges of children with DD in Addis Ababa and on contextual and attitudinal barriers and facilitators for their inclusion in mainstream primary schools.

Methods

We conducted a qualitative study comprising semi-structured interviews with key stakeholders with expertise in DD and/or education in Addis Ababa, the capital city of Ethiopia, from April 2022 to February 2023.

Participants

We planned to interview 8-10 caregivers, 10-15 school representatives (teachers and principals/managers) and 15-10 other professional informants (Non-governmental organisation—NGO—representatives, academics, clinicians, government officials), to ensure a range of roles and perspectives for each participant group.

Caregivers and teachers were recruited from special and mainstream schools using purposive sampling, aiming to ensure that interviewees included caregivers whose children received education in special centers, special units and inclusive classes and, similarly, teachers working in a variety of settings. Caregivers were also recruited from healthcare clinics, with the purpose of seeking caregivers whose children did not attend school. Administration offices at schools and clinics selected by convenience were first contacted as gatekeepers. The informed consent and interviews were conducted by researchers independent of the school administration to ensure voluntary participation. Other stakeholders were recruited through convenience and snowballing sampling, starting by directly contacting stakeholders known to the research team, and subsequently those recommended by interviewees.

Data Collection

Eighteen semi-structured interviews were conducted in English and 21 in Amharic. All caregivers and teachers were interviewed in Amharic, exept for a clinician interviewed in English who is also a primary caregiver. Five interviews in English were conducted virtually, either via phone call or video call, due to practical constraints, while all others happened in person in Addis Ababa.

Interviews consisted of a conversation guided by flexible questions on the meaning of, and value placed on, inclusive education for children with DD, their current educational situation and barriers to education access and quality, and routes to improvement. The interview guide presented variations depending on participant categories, to ensure that everyone's expertise and experience was elicited: for example, caregivers were specifically asked about their child's needs and education experience and teachers about experiences in the classroom. Interview durations varied from 20 minutes to two hours; most interviews lasted 35-55 minutes.

All interviews were audio-recorded and transcribed verbatim, to ensure fidelity to the audio version in the transcription. Transcripts in Amharic were translated to English by an external party.

Demographic data were collected through a brief questionnaire.

Ethical Approval

Ethical approval was obtained from King's College London (RESCM-22/23-21930), the Scientific and Ethical Review Committee at CDT-Africa of Addis Ababa University and the Addis Ababa University College of Health Science Institutional Review Board (061/21/CDT). All participants provided written informed consent to participate.

Analysis

Interview transcripts were analysed using thematic template analysis (Brooks et al., 2014), an approach that is compatible with requirements of the current project: first, co-development of themes in the research team, to ensure multidisciplinary understanding of the data, and Ethiopian as well as outsider perspectives; secondly, the use of an implementation framework to provide structured information for the development and implementation of an intervention.

Four researchers (EG, WB, AC, OB) familiarised themselves with the interview data by transcribing them or reading transcripts, then iteratively developed inductive codes on meaningful sections of the transcripts in NVivo. Each coder independently analysed a subset of the data, after consensus meetings based on initial coding of a few interviews. Once codes were developed for and applied to all interviews, EG organised them deductively in a thematic template based on the Context and Setting dimensions of the Context and Implementation of Complex Interventions (CICI) framework (Pfadenhauer et al., 2017). Throughout these phases, whole-team meetings were held to discuss interpretations and theme development. Participatory stakeholder validation of the analysis was sought by presenting intial themes on contextual factors and eliciting discussion on them at three workshops attended by a variety of stakeholders.

The CICI was chosen for its comprehensive consideration of context, synthesising several previous frameworks, as well as its consideration for how different contextual factors can interact with and affect intervention implementation (Pfadenhauer et al., 2017). Contextual factors are considered at the macro, meso and micro levels and categorised into multiple domains (Pfadenhauer et al., 2017). The geographical context comprises natural and infrastructural environments surrounding implementation. The epidemiological context includes specific features of the target group (in this case, children with DD) and demographics associated with different needs. The socio-economic context concerns the availability of and access to resources and services. The socio-cultural context includes beliefs, common knowledge, patterns of behaviours, cultural customs. The political context describes the distribution of power and the interests of different bodies and individuals holding power. The legal context comprises established laws and rules. The ethical context concerns moral values and principles. Finally, the Setting is a separate CICI dimension, referring to the physical location where an intervention is implemented.

Within themes/domains, subthemes were created inductively from codes, and mapped to macro (whole-country, wider services), meso (within school settings), and micro (relative to the class setting, families or individuals) context levels when relevant to the data.

Positionality

The philosophical stance of the study is critical realism (Bhaskar, 2017): the researchers aimed to investigate an existing reality, and acknowledged that this can only be explored through the different perspectives of stakeholders. In turn, stakeholders' perspectives are interpreted by researchers through their own subjectivity. Consequently, researchers were reflexive of their positionality throughout the study (Braun & Clarke, 2019).

The research team comprises five non-Ethiopian UK-based researchers and students (one based in Ethiopia) and five Ethiopian investigators, including a researcher, a caregiver and special school representative, an academic developmental psychologist, and two clinical academics. All believed in the importance of increasing access to quality education for children with DD.

Of the four researchers who lead the analysis, one is Ethiopian and three identify as outsiders in relation to the Ethiopian population. As such, reflexivity on personal assumptions, and coding discussions between non-Ethiopian and Ethiopian team members were crucial to ground data interpretation in the Ethiopian context.

Results

Participants

We interviewed 39 Ethiopian adult stakeholders (20 women), including 8 teachers (providing mainstream or special education; 3 women), 1 public school principal (0 women), 4 managers, heads or representatives of private inclusive and special schools (2 women), 9 primary caregivers of children with DD (9 women), 6 practicing clinicians (including pediatricians, clinical psychologists and psychiatrists; 5 women), 1 participant who was both a practicing clinician and a primary caregiver (1 woman), 3 local and national government officials (0 women), 3 academics and/or consultants (0 women) and 4 NGO representatives (0 women).

Participants ages ranged from 28 to 65 years. The majority of caregivers were housewives, except for a trader and the practicing clinician mentioned above. Five were married, 2 were single, 2 divorced and 1 widowed. Nine were mothers of the child with DD they cared for and one was an aunt. The ages of the children with DD (4 boys, 6 girls) ranged from 8 to 24, as no limits were imposed when recruiting caregivers. Seven children had an autism diagnosis, at times with co-existing epilepsy and/or intellectual disability; one had a cerebral palsy diagnosis and two a diagnosis of attention-deficit hyperactivity disorder.

Analysis

Below we report our analysis of factors relevant to the Context and Setting dimensions of the CICI framework, for the implementation of interventions to include children with DD in mainstream schools, based on interview data. Quotes are reported verbatim, alongside the participant's code created from a letter ("E" for interviews in English, "A" for interviews in Amharic) and a unique number .

1. **CONTEXT:**

1.1 GEOGRAPHICAL

Discussions on geographical context revolved around infrastructure: specifically, transport and the geographical accessibility of education services for children with DD.

As very few schools (in both the mainstream and special education sector) accept children with DD, they are out of geographical reach for most families. This problem is particularly pronounced for families living in rural areas, where local schools typically do not accept children with DD, due to poorer infrastructure and more negative attitudes towards disabilities. Therefore, these families have to seek enrollment for their children in cities. Even within Addis Ababa, distance creates significant barriers when paired with many challenges in accessing transport.

A024: You can't trust the taxi [minibus, main mean of public transport] and Bajaj [three-wheel taxi] drivers to take her to school, and they are expensive. (caregiver)

1.2 EPIDEMIOLOGICAL

1.2.1 Diversity of needs

Stakeholders highlighted how children with DD are all different, with a range of support needs, which may influence the choice of education setting. Several needs were discussed, including learning difficulties in intellectual and adaptive skills, aggression and other harmful behaviours, hyperactivity, sensory sensitivity needs, communication and social difficulties, and comorbid physical and motor disabilities or medical conditions. Caregivers also reported the different strengths of their children.

A031: Her strengths are that she loves music and works hard. (caregiver)

Multiple professionals and caregivers highlighted the importance of recognising the potential of children with DD: from government official A030 who suggested that, depending on their support needs, children with DD could "become nurses or soldiers", make objects such as "mats and chairs" or "do cleaning jobs", to special school teacher A033, who told the story of the student who "went to Dubai and brought a trophy".

1.2.2 Gender and DD

Interviewers often prompted participants to consider gender influences on inclusion, to account for intersectional exclusion and guide the development of interventions serving boys and girls equally.

Most participants thought boys and girls with DD faced the same challenges in accessing education. A few noted barriers that could disproportionately affect girls. One reported barrier was the inherent exclusion of girls, regardless of disability:

E005: So there is a belief that if a girl is sent to school, she would reach [success] and know her name or... There is a belief in a community like that she couldn't. She can't [have] success in school. She can't reach. She can't go out. She can't work. She can't. For a girl that's general.

Secondly, as the system is more attuned to identify male children with DD and meet their needs, stakeholders reported that girls with DD may not receive the necessary services and accommodations. Thirdly, vulnerability to sexual abuse was a recurrent concern for children with DD in inclusive schools and was reported to disproportionally affect girls:

E013: One of the negative issue is this abuse and the like. The girls could be more affected with that.

1.3 SOCIO-ECONOMIC

1.3.1 Macro socio-economic context

Informants considered Ethiopia’s status as a very low-income country where "all of the projects are donor-driven" (A019, academic) a key contextual factor when considering the inclusion of children with DD in education. An overall lack of resources and services was highlighted. At the same time, stakeholders recognised the valuable role of NGOs and UN organisations and their collaboration with the Ethiopian government.

A036: I think NGOs can do a lot to support inclusive education. They can help children with DD with many things. (teacher)

Beyond the limited access to services, including identification, education, healthcare and employment support services, stakeholders reported a poverty of knowledge resources, specifically of research developments and of appropriate formal training for teachers and special needs education professionals. However, some progress was reported in all these areas.

A019: Believe it or not, we didn’t have any courses on autism until last year. […] The pioneering department in special needs education established it at Addis Ababa University only last year. (academic)

1.3.2 Meso socio-economic context

The lack of economic resources translated into a lack of material and human resources on the ground in school settings. Some private and special schools have resources to provide quality support, but this is only accessible to a limited number of children, generating long waiting lists. At the same time, in government schools, teachers reported not feeling able to address the needs of a child with DD in mainstream classes. They lack special education materials, assistive devices and, most importantly, assistants, as a class of 50-80 students was typically under the responsibility of only one teacher at any given time. Children with DD are therefore turned away, more so than children with physical and sensory disabilities, perceived as having lower support needs. In government schools that accept children with DD, these are cared for in a special unit by a small number of special needs education teachers. Moreover, this profession in under-incentivised and affected by high turn-over.

E006: Being a special need expert or special need teacher, that needs interest, that is commitment and that needs to take everything towards to use. So many teachers are not encouraged to do such things. (government official)

1.3.3 Micro socio-economic context

1.3.3.1: Access to individual support

When children with DD access under-resourced government school settings, they often receive limited support in class, particularly in mainstream classes. Stakeholders discussed how this negatively affects their progress.

E011: He is included in the classrooms, but nobody is assisting him based on his needs. (clinician)

1.3.3.1: Family resources

The overall lack of services also impacts families of children with DD. First, caregivers, often single mothers isolated by their families due to stigma, are unsupported in caring for their children who require constant supervision. In some cases, access to school helps, although it may also create further strain, especially when accompanying the child to school for long distances.

A026: If it had not been for his teachers, I would have had to watch him without doing any work. (caregiver)

A024: It is tiresome. You have to wait in line to get a taxi [public minibus]. It takes me a long to get back home (caregiver)

Secondly, striving to access services for their children generates expenses: paying for access in private well-resourced healthcare and education facilities, buying materials, foregoing work to assist the child in class or hire an assistant.

Overall, stakeholders identified a need to support caregivers financially and psychologically, as well as helping them in tasks such as caring for their children and enrolling them in school.

1.4 SOCIO-CULTURAL

According to stakeholders, social and cultural perspectives at macro, meso and micro levels are mostly characterised by limited awareness about DD and on the needs, rights and strengths of children with DD.

1.4. 1 Macro socio-cultural context

Awareness has spread in the past decade, including through the work of Zemi Yenus, founder of Ethiopia's first school for autistic children, the Joy Center for autism.

A031: Zemi is not alive now, but she has left behind a legacy. Public awareness of autism has increased a lot. (caregiver)

Nonetheless, the lack of awareness in the community and among professionals was still seen as a barrier to identification and recognition, inclusion in society and access to health and education services. Limited awareness took many forms, including spiritual explanations of DD and negative attitudes towards children with DD. Most of all, stakeholders stressed the need to raise awareness "that these children can learn new things, new skills" (E001, clinician) and "that these children can work" (A032, caregiver).

1.4.2 Meso socio-cultural context

Stakeholders identified similar attitudinal barriers within schools, reporting that some principals and teachers display negative attitudes towards children with DD or lack understanding of their needs and potential.

A029: Teachers could punish them because they lack awareness of their condition. [...] They could consider them lazy. (government official)

Additionally, parents of typically developing children can discourage the inclusion of children with DD, by complaining to teachers and principals.

E004: Some parents would come in "I don't want you to put my children/child in that child’s class", or "don't allow him to get next to that child with disability or special needs". (private inclusive school head)

1.4.3 Micro socio-cultural context

1.4.3.1 Interpersonal class context

Negative beliefs and attitudes were also reported among typically developing peers, causing barriers to inclusion within mainstream classes. Relationships with peers were in fact one of the main concerns relative to the inclusion of children with DD in mainstream schools. Specifically, stakeholders reported worries that children with DD may get isolated or bullied by typically developing peers.

E014: If the other kids will not understand the children with limitations, they will abuse them. (caregiver)

1.4.3.2 Family socio-cultural context

Finally, stakeholders reported lack of awareness and negative beliefs within families. Internalised stigma and the belief that children with DD cannot learn can lead caregivers to "withhold their slowly developing children from school" (A022, mainstream class teacher).

1.5 POLITICAL

1.5.1 Macro political context

The limited awareness in the community may also be reflected within Ethiopian institutions, as some political figures "don't know what autism is. They don't know the magnitude of the problem. They don't know what to do." (E008, consultant). Additionally, actions may prioritise the education of typically developing children.

E011: When you tell them that […] they need to be inclusive for kids with intellectual disability and so on, they will tell you that “We're not even providing education for the typical ones”… which is very disappointing that they are, they think that providing education for the typicals [typically developing children] is more important than providing education for the kids with intellectual disability. (clinician)

As such, stakeholders called for the government's attention and commitment to inclusive education, and to services for children with DD more generally. A030 (government official) pointed out that "politics and education should be separated to get out of this problem", to highlight that the commitment to improve education services should not depend on the political beliefs of a given government.

Nonetheless, stakeholders also recognised progress towards building an inclusive society. A019 (academic) and A030 (government official) highlighted how inclusion is a historical process: from initial full exclusion from the society, inclusion is progressing slowly but constantly.

1.5.2 Meso political context

While the government's commitment is necessary to guide country-level progress, the will of each principal is key for ground-level implementation.

A022: The most important thing is a positive attitude from the local community and the school leadership. (mainstream class teacher)

On the other hand, principals’ negative attitudes can cause rejection of children with DD from schools, or a negative environment towards not only children, but even their special education teachers.

A034: Special needs education teachers tell us they face isolation from mainstream classroom teachers. [...] The mainstream teachers themselves look down on special needs education teachers. (special school teacher)

1.6 LEGAL

1.6.1 Macro legal context

Professional stakeholders highlighted the importance of national laws for the education of children with DD. They lamented the existence of fragmented, inconsistent and sometimes outdated policies instead of a cohesive disability policy. Similarly, they reported that the Education and Training policy (1994) was outdated and had little focus on children with disabilities.

Nonetheless, Ethiopia has ratified international policies such as the Salamanca Statement (1994) and the UN CRPD (2006) and subsequently issued national action plans. Such steps reassured professionals of the political commitment to positive change.

A030: The Education and Training policy of Ethiopia was issued in 1994 G.C. before Ethiopia adopted the Salamanca statement, it doesn't include the statement. The policy doesn’t mention anything about inclusive education. However, article 9 of the Ethiopian constitution states that any foreign laws, regulations, and agreements accepted by Ethiopia will be a part of this constitution. (government official)

Several stakeholders stressed that schools are not legally allowed to reject enrollment of children with disabilities. Another positive policy initiative was the establishment of resource centers promoting special needs education in satellite schools through itinerant teachers tasked with supporting other teachers.

However, stakeholders also reflected on some legal systems that pose barriers to the implementation of such action plans. For example, the relative autonomy of regional governments limits the relevance of national policies. Moreover, teachers have little ability to adapt their teaching and dedicate time to children with DD in mainstream classes, due to the limited flexibility of the national curriculum and the need to teach it in time to prepare students for national exams.

Meso legal context

Another reported barrier to inclusion is a policy often adopted by schools, whereby children with DD are only accepted after the family has presented documentation on their disability. Given the challenges in accessing healthcare services, this policy could further delay or stop enrolment.

A026: They refused to accept him if I couldn’t bring his medical report. (caregiver)

1.7 ETHICAL

Stakeholders discussed the value of education for children’s wellbeing, development, and future and its potential to make children with DD independent and productive to the best of their abilities.

A023: These children will serve their country in the future. […] If you don’t teach them, they will become beggars. (mainstream class teacher)

In terms of principles, several professional stakeholders stressed that education was a human right for all children. A minority of them also believed that all children with disabilities had the right to be fully included in mainstream classes and have their needs addressed through inclusive education.

2. SETTING: Relevant features of school buildings

We identified the setting—the physical structure where the intervention takes place—as the school building. Stakeholders reported that most Ethiopian schools are not accessible for children with disabilities. They called for improved infrastructure, ranging from ramps for children with wheelchairs and accessible toilets, to adequate lighting, removal of haphazard from classrooms and playgrounds, and classroom setups which allow for movement and different activities.

E007: Not only always teaching on the chair and table [is best], so maybe they can learn on the the floor, so setting up the floor also is good. (private special school representative)

Haphazard and ineffective school fencing led to safety concerns in inclusive school settings. Such worries discouraged caregivers from seeking enrolment in inclusive schools, where supervision is also perceived as more loose than in special education settings. On their part, principals and teachers were perceived to be often unwilling to take on children with DD and take responsibility for the risks.

A020: He is not the kind of child that you can manage. What if they enroll him in the school and he escapes their watch and gets hit by a car? (caregiver)

Discussion

In this study, we interviewed 39 stakeholders and mapped their reports to domains in the Context and Setting dimensions of the CICI framework (Pfadenenhauer et al., 2017), to explore contextual factors relevant to including children with DD in Ethiopian mainstream schools, with a focus on Addis Ababa. Our data showed that, while general awareness of DD may have increased compared to previous evidence (Tekola et al., 2020; Tilahun et al., 2016), children with DD are still largely excluded from education. Several contextual barriers reported from specific school settings and family contexts up to the the national level were comparable to those highlighted in other African countries (Genovesi et al., 2022).

The right of children with DD to inclusion in education was highlighted by various professionals referring to both moral and legal frameworks. The latter, for example the Education Sector Development Programmes

(Federal Democratic Republic of Ethiopia Ministry of Education 2015; 2021) were praised as showing the government's commitment to inclusion. However, some stakeholders also reported the lack of focus on children with disabilities in the national Education and Training Policy (Federal Democratic Republic of Ethiopia Ministry of Education, 1994). In practice, as highlighted by previous findings, the availability of special and mainstream schools that accept children with DD is overall limited (Tekola et al., 2016). In mainstream schools, limited awareness among staff and communities at large plays a role in such rejections. Stakeholders reported a substantial increase in community awareness of DD in recent years and multiple examples of small- and large-scale awareness-raising efforts. However, our findings show a remaining need to reduce negative attitudes towards children with DD and raising awareness of their ability to learn. Moreover, we identified geographical accessibility as an important barrier: the few special and mainstream schools accepting children with DD often serve large areas, with families coming from far away and struggling with the economic and time costs of transportation. These challenges enhance the burden of care placed on caregiver, often isolated mothers (Side & Kumar, 2022; Szlamka et al., 2023; Tekola et al., 2022). On the other end, when these struggles are removed, being able to take their child to school reduces the burden on the caregiver, as the school shares the caregiving responsibility. Consequently, making each local school an appropriate setting for the enrolment of children with DD would ensure that they can access education with few struggles, upholding their rights and supporting their caregivers.

Stakeholders also discussed several systematic barriers previously highlighted with regards to including children with DD in mainstream schools in other African countries (Genovesi et al., 2022) and children with disabilities more generally in Ethiopia (Beyene & Tizazu, 2010; Franck & Joshi, 2017; Ginja & Chen, 2021; Schiemer, 2017): limited material and human resources, lack of specialised training and teacher support, limited curriculum flexibility, and poorly accessible infrastructure. Burningham, Chen, Genovesi et al. (2023) analyse in-depth how these barriers can negatively affect children in DD in mainstream schools, causing concerns about their development and learning as well as their safety. In the current analysis we have reported that these barriers also act prior to children with DD being enrolled in schools. For example, teachers who have to manage classes of up to 80 children may reject enrolment of children with DD because they feel unable to take responsibility for their supervision and safety, or to appropriately support their learning. Their limited training in special needs and duty to teach a rigid curriculum further lower their confidence in their ability to meet the needs of children with DD. Therefore, an implementation plan for including children with DD must address these barriers, so that children with DD can both access school and benefit from it.

Limitations

Eighteen of the 39 interviews were conducted by the first author in English, which was not the native language of the respondents. Interviews in English allowed the first author to immerse herself in the context and gain a deeper understanding on the data. The resulting reports may slightly diverge from the ones that participants would have provided in their mother tongue—Amharic. However, all the 18 participants were professionals with good conversational English skills and the impact of this limitation on the findings is unlikely to be substantial. The remaining interviews were conducted in Amharic by native Amharic speakers.

Additionally, children with DD who are identified in Ethiopia are typically those with high support needs and substantial cognitive and communication difficulties and we were not able to interview them directly. While

caregivers were asked about their children’s experiences and some professional participants had lived experience of disability, the fact that children with DD themselves were not interviewed is a limitation of this study.

Conclusion

Political, legal, and moral frameworks in Ethiopia recognise all children’s right to education. However, children with DD in Addis Ababa currently have limited opportunities to access education, either in special schools or the few mainstream schools that accept their enrolment. Sociocultural and socioeconomic barriers, including limited awareness of DD and limited material, human and training resources, hinder their inclusion. Our systematic, in-depth analysis of contextual and setting factors can form the basis for development of an implementation model that addresses such barriers and capitalises on existing facilitators.

Acknowledgements

We would like to thank all participants. We also thank Mr Mersha Kinfe and staff at the Addis Ababa University Center for Innovative Drug Development and Therapeutic Trials for Africa (CDT-Africa) for their support in data collection.

References

Andrews, C., Kakooza-Mwesige, A., Almeida, R., Swartling Peterson, S., Wabwire-Mangen, F., Eliasson, A. C., & Forssberg, H. (2020). Impairments, functional limitations, and access to services and education for children with cerebral palsy in Uganda: a population-based study. *Developmental Medicine & Child Neurology*, *62*(4), 454-462. https://doi.org/10.1111/dmcn.14401

Beyene, G., & Tizazu, Y. (2010). Attitudes of teachers towards inclusive education in Ethiopia. *Ethiopian Journal of Education and Sciences*, *6*(1). https://doi.org/10.4314/ejesc.v6i1.65383

Burningham, O., Chen, A., Genovesi, E., Belay, W., Ahmed, I., Ayele, M., … Hoekstra, R. (2023, November 2). Inclusive Education for Children with Developmental Disabilities in Ethiopia: Stakeholder Views on Benefits, Disadvantages and Priorities for Action. *PsyArXive Preprints*. https://doi.org/10.31234/osf.io/v6snh

Bhaskar R. (2017) The order of natural necessity: a kind of introduction to critical realism. London: Gary Hawke; 2017.

Braun, V., & Clarke, V. (2019). Reflecting on reflexive thematic analysis. *Qualitative Research in Sport, Exercise and Health*, *11*(4), 589–597. https://doi.org/10.1080/2159676X.2019.1628806

Burningham, O., Chen, A., Genovesi, E., Belay, W., Ahmed, I., Ayele, M., … Hoekstra, R. (2023). Inclusive Education for Children with Developmental Disabilities in Ethiopia: Stakeholder Views on Benefits, Disadvantages and Priorities for Action. *PsyArXive Preprints*. https://doi.org/10.31234/osf.io/v6snh

Colombi, C., & Ghaziuddin, M. (2017). Early Intervention for Children With Autism Spectrum Disorder in Low-Resource Countries. *J. Am. Psychiatr. Nurses Assoc*, *23*, 344-345. https://doi.org/10.1177/1078390317717329

Federal Democratic Republic of Ethiopia Ministry of Education. (1994). Education and Training Policy. Addis Ababa, Ethiopia: Ministry of Education

Federal Democratic Republic of Ethiopia Ministry of Education. (2015a). *A Master plan for special needs education/inclusive education in Ethiopia 2016-2025*. https://planipolis.iiep.unesco.org/en/2016/master-plan-special-needs-educationinclusive-education-ethiopia-2016-2025-6465

Federal Democratic Republic of Ethiopia Ministry of Education. (2015b). *Education Sector Development Programme V 2015/16-2019/20.* https://www.google.com/search?client=safari&rls=en&q=FDR+Ethiopia+Ministry+of+Education.+Education+Sector+Development+Programme+V+2015/16-2019/20+(2015)&ie=UTF-8&oe=UTF-8

Federal Democratic Republic of Ethiopia Ministry of Education. (2020). *Education Sector Development Programme VI 2013-2017 E.C. 2020/21-2024/25.* https://assets.globalpartnership.org/s3fs-public/document/file/2021-11-education-sector-development-plan-ethiopia.pdf?VersionId=eCE8EO7S11XRa806tewLkhRcQfQ6yU2B

Franck B. & Joshi, D.K. (2017). Including students with disabilities in Education for All: lessons from Ethiopia. *International Journal of Inclusive Education, 21*(4), 347-360, https://doi.org/10.1080/13603116.2016.1197320

Genovesi, E., Jakobsson, C., Nugent, L., Hanlon, C., & Hoekstra, R. A. (2021). Stakeholder experiences, attitudes and perspectives on inclusive education for children with developmental disabilities in

sub-Saharan Africa: A systematic review of qualitative studies. *Autism*, 13623613221096208. https://doi.org/10.1177/13623613221096208

Genovesi, E., Ayele, M., Ahmed, I., Belay, W., Burningham, O., Chen, A., … Hoekstra, R. (2023, November 2). Developing an implementation model for the inclusion of children with developmental disabilities in mainstream primary schools in Ethiopia. *PsyArXive Preprints*. https://doi.org/10.31234/osf.io/2g3eq

Ginja, T. G., & Chen, X. (2021). Conceptualising inclusive education: the role of teacher training and teacher's attitudes towards inclusion of children with disabilities in Ethiopia. *International Journal of Inclusive Education*, *27*(9), 1042-1055.https://doi.org/10.1080/13603116.2021.1879958

Mergia, A.T. (2020). The practice of inclusive education in Ethiopia. *UNESCO Digital Library*. https://unesdoc.unesco.org/ark:/48223/pf0000373678

Olusanya, B. O., Davis, A. C., Wertlieb, D., Boo, N. Y., Nair, M. K. C., Halpern, R., ... & Kassebaum, N. J. (2018). Developmental disabilities among children younger than 5 years in 195 countries and territories, 1990–2016: a systematic analysis for the Global Burden of Disease Study 2016. *The Lancet Global Health*, *6*(10), e1100-e1121. https://doi.org/10.1016/S2214-109X(18)30309-7

Padeliadu, S. & Lampropolou, V. (1997). Attitudes of Special and regular education teacher towards school integration. *European journal of Special needs education. 12*(3), 173-183. Cited by Beyene, G., & Tizazu, Y. (2010). Attitudes of teachers towards inclusive education in Ethiopia. *Ethiopian Journal of Education and Sciences*, *6*(1). https://doi.org/10.4314/ejesc.v6i1.65383

Pfadenhauer, L.M., Gerhardus, A., Mozygemba, K., Lysdahl, K.B., Booth, A., Hofmann, B., ... Rehfuess, E. (2017). Making sense of complexity in context and implementation: the Context and Implementation of Complex Interventions (CICI) framework. *Implementation Science*, *12*(1), 1-17. https://doi.org/10.1186/s13012-017-0552-5

Price, R. (2018). Inclusive and special education approaches in developing countries. K4D Helpdesk Report 373. Brighton, UK: Institute of Development Studies. [cited on 19th August 2023] Retrieved from: https://opendocs.ids.ac.uk/opendocs/handle/20.500.12413/14284

Side, A.S. & Kumar, R.S. (2022). Stressors and Coping Strategies among Parents of Children with Autism Spectrum Disorder in Ethiopia. *Bahir Dar Journal of Education, 22*(1), 76-96. Retrieved from https://www.ajol.info/index.php/bdje/article/view/239894

Schiemer, M. (2017). *Education for children with disabilities in Addis Ababa, Ethiopia: Developing a sense of belonging* (p. 200). Springer Nature. https://doi.org/10.1007/978-3-319-60768-9

Šiška, J., Bekele, Y., Beadle-Brown, J., Záhořík, J. (2020). Role of resource centres in facilitating inclusive education: experience from Ethiopia. *Disability & Society, 35*(5), 811-830. https://doi.org/10.1080/09687599.2019.1649120

Szlamka, Z., Ahmed, I., Genovesi, E., Kinfe, M., Hoekstra, R.A., Hanlon, C. (2023). Conceptualising the empowerment of caregivers raising children with developmental disabilities in Ethiopia: a case study. OSF. https://doi.org/10.17605/OSF.IO/SRC5W

Tekola, B., Baheretibeb, Y., Roth, I., Tilahun, D., Fekadu, A., Hanlon, C., & Hoekstra, R. A. (2016). Challenges and opportunities to improve autism services in low-income countries: lessons from a situational analysis in Ethiopia. *Global Mental Health*, *3*. https://doi.org/10.1017/gmh.2016.17

Tekola, B., Girma, F., Kinfe, M., Abdurahman, R., Tesfaye, M., Yenus, Z., ... & Hoekstra, R. A. (2020). Adapting and pre-testing the World Health Organization's Caregiver Skills Training programme for autism and other developmental disorders in a very low-resource setting: Findings from Ethiopia. *Autism*, *24*(1), 51-63. https://doi.org/10.1177/1362361319848532

Tekola, B., Kinfe, M., Girma Bayouh, F., Hanlon, C., & Hoekstra, R. A. (2023). The experiences of parents raising children with developmental disabilities in Ethiopia. *Autism*, *27*(2), 539-551. https://doi.org/10.1177/13623613221105085

Tesfaye A. (2005). The attitudes of special and regular class teacher towards the inclusion individuals with mental retardation. Unpublished Masters Thesis. Addis Ababa. AAU press. Cited by Beyene, G., & Tizazu, Y. (2010). Attitudes of teachers towards inclusive education in Ethiopia. *Ethiopian Journal of Education and Sciences*, *6*(1). https://doi.org/10.4314/ejesc.v6i1.65383

Tilahun, D., Hanlon, C., Fekadu, A., Tekola, B., Baheretibeb, Y., & Hoekstra, R. A. (2016). Stigma, explanatory models and unmet needs of caregivers of children with developmental disorders in a low-income African country: a cross-sectional facility-based survey. *BMC health services research*, *16*(1), 1-12. https://doi.org/10.1186/s12913-016-1383-9

UN Committee on the Rights of Persons with Disabilities (Sixteenth session). (2016a). *Summary record (partial) of the 271st meeting: Consideration of reports submitted by parties to the Convention under article 35 (continued), Initial report of Ethiopia (continued).* United Nations Digital library. https://digitallibrary.un.org/record/1314826

UN Committee on the Rights of Persons with Disabilities (Sixteenth session). (2016b). *Summary record (partial) of the 272nd meeting: Consideration of reports submitted by parties to the Convention under article 35 (continued), Initial report of Ethiopia (continued).* United Nations Digital library. https://digitallibrary.un.org/record/1314826

UN General Assembly. (2007). *Convention on the Rights of Persons with Disabilities (CRPD).* United Nations Enable.

Zeleke, W.A., Hughes, T., & Chitiyo, M. (2018). The path to an autism spectrum disorders diagnosis in Ethiopia: Parent perspective. *American Journal of Orthopsychiatry, 88*(3), 316–327. https://doi.org/10.1037/ort0000249

Zerihun, T., Kinfe, M., Koly, K. N., Abdurahman, R., Girma, F., WHO Team, ... & Hoekstra, R. A. (2023). Non-specialist delivery of the WHO Caregiver Skills Training Programme for children with developmental disabilities: Stakeholder perspectives about acceptability and feasibility in rural Ethiopia. *Autism*, 13623613231162155. https://doi.org/10.1177/13623613231162155
