# Supplementary material for: Exploring context for implementation of inclusive education for children with developmental disabilities in mainstream primary schools in Ethiopia
Source: PLoS One. 2024 Aug 9;19(8):e0307576. doi: 10.1371/journal.pone.0307576 (PMC11315310; doi:10.1371/journal.pone.0307576)
Supplement: Attachment — Submitted filename: Response to Reviewers.docx [file pone.0307576.s004.docx]

**8^th^ February 2024**

**Re: Revision of Manuscript Submission PONE-D-23-36308**

Dear Dr Mulu Tiruneh,

Thank you for your kind feedback and your valuable suggestions on our manuscript "Exploring context for implementation of inclusive education for children with developmental disabilities in mainstream primary schools in Ethiopia".

We are resubmitting the manuscript, that now meets PLOSONE style requirements and includes a correct and complete financial disclosure. We have attended to each comment made by the editor and the reviewers and believe the recommended revisions have strengthened our manuscript. In this letter, we provide a point-by-point response to the editors’ and each reviewer’s comments. The responses, initially submitted on 6^th^ February, have been updated following the journal’s communication of 7^th^ February.

The manuscript includes tracked changes of revisions, while a few subheading revisions are not tracked but highlighted.

We hope that the revisions are to your satisfaction and look forward to hearing from you.

Yours sincerely, on behalf of all co-authors,

Elisa Genovesi

**Response to the editor, in relation to journal requirements**

**1. When submitting your revision, we need you to address these additional requirements. Please ensure that your manuscript meets PLOS ONE's style requirements, including those for file naming.**

We would like to thank the editorial board for the waiving of formatting requirements at the point of submission and for the clear guidelines provided for resubmission. We have revised the formatting based on PLOSONE guidelines, including those for file names.

**2. Did you know that depositing data in a repository is associated with up to a 25% citation advantage (**[**https://doi.org/10.1371/journal.pone.0230416**](https://doi.org/10.1371/journal.pone.0230416)**)? If you’ve not already done so, consider depositing your raw data in a repository to ensure your work is read, appreciated and cited by the largest possible audience. You’ll also earn an Accessible Data icon on your published paper if you deposit your data in any participating repository (**[**https://plos.org/open-science/open-data/#accessible-data**](https://plos.org/open-science/open-data/#accessible-data)**).**

Please see point 6.

**3. We note that the grant information you provided in the ‘Funding Information’ and ‘Financial Disclosure’ sections do not match.**

**When you resubmit, please ensure that you provide the correct grant numbers for the awards you received for your study in the ‘Funding Information’ section.**

Thank you for pointing out the error, please find the up to date funding information below. This was removed from the manuscript and reported here as requested in the communication of 07/02/2024.

“The project has been funded by the UKRI Economic and Social Research Council through a London Interdisciplinary Social Science Doctoral Training Partnership (LISS DTP) studentship (ES/P000703/1), by the British Institute for Eastern Africa through a Thematic Grant and by undergraduate research project funding from King’s College London. CH receives support from the Wellcome Trust through grants 222154/Z20/Z and 223615/Z/21/Z. RAH, CH, FG and WB receive support from the National Institute for Health and care Research (NIHR200842) using UK aid from the UK Government and CH through the NIHR Global Health Research Group on Homelessness and Mental Health in Africa (NIHR134325) using UK aid from the UK Government. The views expressed in this publication are those of the authors and not necessarily those of the NIHR or the Department of Health and Social Care or other funders. The funders had no role in study design, data collection and analysis, decision to publish, or preparation of the manuscript.”

**4. Thank you for stating the following financial disclosure: 
The project has been funded by the UKRI Economic and Social Research Council through a London Interdisciplinary Social Science Doctoral Training Partnership (LISS DTP) studentship (ES/P000703/1), by the British Institute for Eastern Africa through a Thematic Grant and by undergraduate research project funding from King’s College London. RAH, CH, FG and WB receive support from the National Institute for Health and care Research (NIHR200842) using UK aid from the UK Government and CH through the NIHR Global Health Research Group on Homelessness and Mental Health in Africa (NIHR134325) using UK aid from the UK Government. The views expressed in this publication are those of the authors and not necessarily those of the NIHR or the Department of Health and Social Care or other funders. CH receives support from the Wellcome Trust through grants 222154/Z20/Z and 223615/Z/21/Z.**

**Please state what role the funders took in the study.  If the funders had no role, please state: "The funders had no role in study design, data collection and analysis, decision to publish, or preparation of the manuscript." 
If this statement is not correct you must amend it as needed. 
Please include this amended Role of Funder statement in your cover letter; we will change the online submission form on your behalf.**

Thank you, the sentence above has been added to the financial statement reported above.

**5. We noted in your submission details that a portion of your manuscript may have been presented or published elsewhere. The results in this manuscript have not been published, nor are under consideration anywhere else, however there are two additional studies using the same dataset for two clearly distinct analyses. The study produced a large dataset, as topic guides for stakeholder interviews explored three different areas: (a) stakeholders own views of how inclusive education could benefit or disadvantage children with developmental disabilities in Ethiopia, (b) the current education provision for children with developmental disabilities in the country and barriers and facilitators for inclusion, including for teachers, principals, caregivers and other stakeholders, (c) recommended implementation processes and strategies for effective inclusion of children with developmental disabilities in mainstream schools. These three distinct research questions were answered through three analyses with theme development processes independent from each other.
The enclosed manuscript reports analysis and its companion manuscript that reports analysis will be submitted to PLOS ONE shortly after this. The two are companions as they both use the same implementation framework for theme development, however the enclosed manuscript focuses on the Context and Setting domains only and the other on Implementation only. As such, results are complementary but often refer to different data extracts and are entirely relevant to different topics, rather than two parts of the same manuscript. The two manuscripts can be read independently from each other.
The manuscript reporting analysis, under consideration elsewhere, focuses exclusively on outcomes for children with developmental disabilities, ignoring broader contextual and implementation factors and processes relevant to effective inclusion. Such manuscript represents a background analyses to the enclosed manuscript: it reports that stakeholders consider inclusion in mainstream schools as a potentially beneficial practice for children with developmental disabilities. Such finding is not directly relevant to the research question of analysis, and rather is an important background consideration prior to exploring the readiness of the context for implementing such change, as the enclosed manuscript aims to do. Please clarify whether this [conference proceeding or publication] was peer-reviewed and formally published. If this work was previously peer-reviewed and published, in the cover letter please provide the reason that this work does not constitute dual publication and should be included in the current manuscript.**

Thank you for the opportunity to explain this aspect. We would like to clarify that no portion of the manuscript is under consideration elsewhere. Two other manuscripts on related but distinct topics are under consideration for publication in peer-reviewed journals; one of the two is under consideration for publication in PLOSONE itself. This work does not constitute dual publication because the manuscripts explore distinct research questions and report different analyses conducted with different codes on different portions of the data. Such portions of the data come from the same set of interviews simply because the three research topics are related and it would have been impractical to ask questions relative to these topics in three separate sets of interviews.

**6. In the online submission form, you indicated that Data will be available upon request. The authors have carefully considered making the data available through a repository. However, in qualitative research and in global health this poses a series of issues, also recognised by evidence (e.g. Tsai et al., 2016). As such, the authors have agreed that it would be unethical to make data publicly available. First, quotes is the manuscript are carefully selected so as to not identify participants or link them to their personal data, but full transcripts may identify them, as professionals working on developmental disabilities in Addis Ababa are few and known to the population. The trascripts may also include personal data beyond participants' perspectives, such as their diagnosis or that of their children. Secondly, the data would largely not be available to researchers in Africa, who are disadvantaged when it comes to accessing publicly available data. Instead, it may be accessible to researchers in high-income countries who may be unfamiliar with the context, leading to potentially harmful interpretations of the data.**

**All PLOS journals now require all data underlying the findings described in their manuscript to be freely available to other researchers, either a. In a public repository, b. Within the manuscript itself, or c. Uploaded as supplementary information.
This policy applies to all data except where public deposition would breach compliance with the protocol approved by your research ethics board. If your data cannot be made publicly available for ethical or legal reasons (e.g., public availability would compromise patient privacy), please explain your reasons on resubmission and your exemption request will be escalated for approval.**

Thank you for the opportunity to explain this aspect. Indeed, our manuscript fits in the exceptions to the policy, as in our case public deposition would breach compliance with the protocol approved by our research ethics boards. In the approved consent forms, participants agreed for the data to be shared with Ethiopia-based researchers, but consent for public deposition was not obtained. Moreover, the information sheets, consent forms and protocol approved by our research ethics boards specify that any use of the data will ensure that participants cannot be identified in any outputs. As previously described, the nature of our data (including quotes within transcripts themselves) means that the latter cannot be granted when making the data publicly available, resulting in a breach of the approved protocol and consent obtained.

A further communication from the journal on 07/02/2024 reads “This policy applies to all data except where public deposition would breach compliance with the protocol approved by your research ethics board. If your data cannot be made publicly available for ethical or legal reasons (e.g., public availability would compromise patient privacy), please explain your reasons on resubmission and your exemption request will be escalated for approval.”

As explained above, indeed in our case “public deposition would breach compliance with the protocol approved” by our research ethics boards. Public deposition of the transcripts would breach the anonymity of participants and be available to research teams with no connections to African countries. For these reasons, during the ethical review process, which was rigorously conducted by three review boards, one at King’s College London and two at Addis Ababa University, we were unable to obtain approval for public deposition and to include data deposition in our Consent Form for participation. Therefore, publicly depositing the data would breach ethical approvals and participants’ informed consent.

**7. We note that you have referenced Tesfaye A. (2005).  which has currently not yet been accepted for publication. Please remove this from your References and amend this to state in the body of your manuscript: (Tesfaye A. (2005). [Unpublished]”) as detailed online in our guide for authors**
[**http://journals.plos.org/plosone/s/submission-guidelines#loc-reference-style**](http://journals.plos.org/plosone/s/submission-guidelines#loc-reference-style)

Thank you for these guidelines that we have now met.

**8. We note that you have referenced UN Committee on the Rights of Persons with Disabilities (Sixteenth session). (2016a) which has currently not yet been accepted for publication. Please remove this from your References and amend this to state in the body of your manuscript: (UN Committee on the Rights of Persons with Disabilities (Sixteenth session). (2016a). [Submitted]”) as detailed online in our guide for authors**
[**http://journals.plos.org/plosone/s/submission-guidelines#loc-reference-style**](http://journals.plos.org/plosone/s/submission-guidelines#loc-reference-style)

**&**

**9. We note that you have referenced UN Committee on the Rights of Persons with Disabilities (Sixteenth session). (2016b) which has currently not yet been accepted for publication. Please remove this from your References and amend this to state in the body of your manuscript: (UN Committee on the Rights of Persons with Disabilities (Sixteenth session). (2016b). [Submitted]”) as detailed online in our guide for authors**
[**http://journals.plos.org/plosone/s/submission-guidelines#loc-reference-style**](http://journals.plos.org/plosone/s/submission-guidelines#loc-reference-style)

The documents mentioned above are policy documents, published as UNESCO records at <https://digitallibrary.un.org/record/1314826>. These references have now been corrected and are included in the reference list as follows:

14. UN Committee on the Rights of Persons with Disabilities. Summary record (partial) of the 271st meeting, held at the Palais Wilson, Geneva, on Tuesday, 16 August 2016 :: Committee on the Rights of Persons with Disabilities, 16th session. 2016 Aug 24 [cited 2024 Feb 6]; Available from: https://digitallibrary.un.org/record/1316834

15. UN Committee on the Rights of Persons with Disabilities. Summary record (partial) of the 272nd meeting, held at the Palais Wilson, Geneva, on Wednesday, 17 August 2016 :: Committee on the Rights of Persons with Disabilities, 16th session. 2016 Aug 24 [cited 2024 Jan 31]; Available from: https://digitallibrary.un.org/record/1314826

**Response to comments of Reviewer 1**

**It is very interesting article.I like so much! The manuscript is technically sound able and it has great potential. As is is qualitative study I didn't expect special statistical techniques and it is written in a well organized way.**

Thank you for your feedback, we are glad you appreciated our work!

**Response to comments of Reviewer 2**

**Thank you for the opportunity to review this manuscript. I've included some suggestions and comments.**

Thank you for your feedback and suggestions. Please see our responses to your comments below.

**General comment 1. Please use Line Numbers next time to facilitate the review.**

**&**

**C. Materials and Methods: 1. It is ‘Materials and Methods’ not ‘Methods’, based on guidelines.**

**&**

**F. References: 1. Please use the right style of in-text referencing and bibliography.**

Thank you for these guidelines. PLOSONE kindly waives formatting requirements at first submission. We have now formatted the manuscript for resubmission and met the recommendations above.

**General comment 2. Please ensure that the headings and subheadings are numbered consistently. For example, Materials and Methods section were no numbered. Some numbers of subheading are duplicated (e.g. 1.3.3.1).**

Thank you. We have now corrected the error and included a thematic template at page 9 for further clarity.

**3. It is recommended to upload the interview guideline and data analysis (coding).**

Thank you for this suggestion. Both are now available as supplementary files.

**A. Title: 1. Authors should use ‘Addis Ababa, Ethiopia’ instead of ‘Ethiopia’ only which may limit the generalizability of the findings.**

Thank you for this suggestion that has helped us better explain our aims in the manuscript. However, we would like to keep the original title. The flexibility provided by qualitative research methods allowed us to ask questions on both Addis Ababa and Ethiopia overall in our interviews, as we have now clarified at lines 36, 42, 107, 126 and 565-567. As such, our data reflects the general situation of Ethiopian mainstream schools, with a focus on Addis Ababa, but also several references to rural area (as mentioned for example at line 265). This variability does not impact the quality and trustworthiness of our data, as concepts of generalisability and representativeness are not applicable to qualitative research and we have described in the Results when the data refer to Addis Ababa, other areas, or Ethiopia in general.

**B. Introduction:**

**1. Could you please let us know the latest statistical data on the number of children with DD in Ethiopia?**

To the best of our knowledge, unfortunately such data are not available.

**2. In the authors' second paragraph, they stated that services for children with developmental disabilities (DD) are limited, and that there are unmet needs in accessing professional support for these children. Can you first provide an example of the types of services and professional support that are DD children need?**

Thank you for this suggestion. Lines 55-65 have now been edited as follows: “They are a substantial cause of disability globally and characterised by high support needs (Colombi & Ghaziuddin, 2017; Olusanya et al., 2018). Children with DD may need a range of healthcare and educational services to support their cognitive, social and motor development, including the assistance of mental health and rehabilitation specialists. However, these services are often limited, particularly in low- and middle-income countries, where most children with DD reside (Olusanya et al., 2018).

In Ethiopia, a very low-income country in Eastern Africa, diagnostic, healthcare, educational and rehabilitation services for children with DD are limited (Tekola et al., 2016) and often unknown to families who need them (Zeleke et al., 2018).”

**C. Materials and Methods**

**2. I wonder how semi-structured interviews guideline was developed, validated, and relied?**

Information on development and revisions of the topic guide has now been provided at lines 157-159. Topic guides for semi-structured interviews are flexible entities that aim to obtain qualitative rather than quantitative data and as such no validity and reliability measures were needed or possible.

**3. This part should be in Materials and Methods section not in results.**

**‘Analysis: Below we report our analysis of factors relevant to the Context and Setting dimensions of the CICI framework, for the implementation of interventions to include children with DD in mainstream schools, based on interview data. Quotes are reported verbatim, alongside the participant’s code created from a letter (“E” for interviews in English, “A” for interviews in Amharic) and a unique number’ .**

We have moved this paragraph it to lines 199-201 and amended it to fit in this section.

**D. Results: 1. I would prefer to summarise your participants in a table then report it.**

Thank you for this suggestion. We have now summarised the most complex parts of the information in Tables 1 and on pages 8 and 9.

**E. Discussion: 1. It wasn't enough discussion. Further divided into concise subsections based on outcomes.**

Thank you for this comment. We have now significantly extended the discussion, by highlighting our results with one or more concise sentences for each theme at lines 527-555, as reported below:

“In Theme 1 (Geographical context), we discussed reported challenges with reaching inclusive schools, especially but not only for families living in rural areas, due to their distance and the lack of inclusive public transport. In the first subtheme (Diversity of needs) of Theme 2 (Epidemiological context) we reported stakeholders’ discourses on the diverse range of needs and strengths among children with DD and the importance of recognising their potentials. The second subtheme (Gender and DD) concerned child features that may affect their inclusion. Specifically, a small but substantial minority of stakeholders believed that girls with DD may be more excluded than boys, due to the inferior value placed on girls’ education in general and to sexual abuse concerns. In Theme 3 (Socio-economic context), we presented stakeholders’ reports of various economic and service access challenges that from the macro, or national, level—specifically Ethiopia’s low-income status and reliance on external donors—reflect on the meso level—with a lack of material and human resources in schools—and the micro level—with the poverty of many families of children with DD and ultimately the lack of access to individualised support for children themselves. Theme 4 (Socio-cultural context) discusses attitudinal barriers to the inclusion of children with DD, specifically the limited awareness and stigma nationally, among principals, teachers, typically developing children and their parents within school communities and even internalised within families of children with DD. Socio-cultural barriers were reported as decreasing, but still key, and bullying among children was especially a concern for inclusion. In Theme 5 (Political context), the first subtheme (Macro political context) concerns stakeholders’ calls for greater political commitment at the national level. The second subtheme (Meso political context) discusses the importance of school principals’ commitment to inclusion, equally reported as crucial by stakeholders. Similarly, in Theme 6 (Legal context), the first subtheme (Macro legal context) concerns national policies and the second (Meso legal context) school regulations. Stakeholders described national policies as fragmented, inconsistent and sometimes outdated, while also praising the ratification of relevant international policies and subsequent national action plans. A reported barrier in school regulations is the requirement for a disability certificate for school enrolment, which may delay enrolment itself. Theme 7 (Ethical context) describes relevant ethical values in Ethiopia, specifically the value placed on the education of all children as a human right and to promote their future contribution to the society. Finally, in Theme 8 (Setting) we discussed school building features as the implementation setting for inclusion, and highlighted stakeholders’ concerns for the scarce accessibility and safety of most school buildings.”

Lines 556-564, which are the beginning of the more organic discussion of the themes in relation to each other and the wider literature, have been adapted to accommodate the above change.
