# Supplementary material for: Exploring context for implementation of inclusive education for children with developmental disabilities in mainstream primary schools in Ethiopia
Source: PLoS One. 2024 Aug 9;19(8):e0307576. doi: 10.1371/journal.pone.0307576 (PMC11315310; doi:10.1371/journal.pone.0307576)
Supplement: Attachment — Submitted filename: Response to Reviewers.docx [file pone.0307576.s005.docx]

**27^th^ June 2024**

**Re: Revision of Manuscript Submission PONE-D-23-36308**

Dear Dr Mulu Tiruneh,

Thank you for kindly advancing the review process for our manuscript "Exploring context for implementation of inclusive education for children with developmental disabilities in mainstream primary schools in Ethiopia" and conveying the comment offered by Reviewer 1 in this second round of reviews.

In the manuscript we are now submitting, we have simplified language at lines 190-192, based on the reviewer’s comment that there was “No need of saying in such scientific writing”, which we agree with and appreciate.

We hope that the revision is to your satisfaction and look forward to hearing from you.

Yours sincerely, on behalf of all co-authors,

Elisa Genovesi
